# How Schools Affect Student Well-Being: A Cross-Cultural Approach in 35 OECD Countries

**DOI:** 10.3389/fpsyg.2020.00431

**Published:** 2020-03-25

**Authors:** Elena Govorova, Isabel Benítez, José Muñiz

**Affiliations:** ^1^Department of Psychology, University of Oviedo, Oviedo, Spain; ^2^Department of Methodology of Behavioural Sciences, University of Granada, Granada, Spain

**Keywords:** well-being, school effectiveness, Programme for International Student Assessment, science, hierarchical linear modeling

## Abstract

A common approach for measuring the effectiveness of an education system or a school is the estimation of the impact that school interventions have on students’ academic performance. However, the latest trends aim to extend the focus beyond students’ acquisition of knowledge and skills, and to consider aspects such as well-being in the academic context. For this reason, the 2015 edition of the international assessment system Programme for International Student Assessment (PISA) incorporated a new tool aimed at evaluating the socio-emotional variables related to the well-being of students. It is based on a definition focused on the five dimensions proposed in the PISA theoretical framework: cognitive, psychological, social, physical, and material. The main purpose of this study is to identify the well-being components that significantly affect student academic performance and to estimate the magnitude of school effects on the well-being of students in OECD countries, the school effect being understood as the ability of schools to increase subjective student well-being. To achieve this goal, we analyzed the responses of 248,620 students from 35 OECD countries to PISA 2015 questionnaires. Specifically, we considered non-cognitive variables in the questionnaires and student performance in science. The results indicated that the cognitive well-being dimension, composed of enjoyment of science, self-efficacy, and instrumental motivation, as well as test anxiety all had a consistent relationship with student performance across countries. In addition, the school effect, estimated through a two-level hierarchical linear model, in terms of student well-being was systematically low. While the school effect accounted for approximately 25% of the variance in the results for the cognitive dimension, only 5–9% of variance in well-being indicators was attributable to it. This suggests that the influence of school on student welfare is weak, and the effect is similar across countries. The present study contributes to the general discussion currently underway about the definition of well-being and the connection between well-being and achievement. The results highlighted two complementary concerns: there is a clear need to promote socio-emotional education in schools, and it is important to develop a rigorous framework for well-being assessment. The implications of the results and proposals for future studies are discussed.

## Introduction

The effectiveness of an education system or a school is generally measured in terms of the impact that school interventions have on student performance, with the prevalent focus being on the cognitive elements, and mostly those associated with the requirements of the academic curriculum or competence areas. Following the definition of Murillo (2005), a school is considered effective when it achieves the maximum holistic development of every one of its students, and especially when this development is greater than might be expected considering the student’s previous performance and/or the social, economic, and cultural situation of his/her family. Although a student’s development is expected to be comprehensive, school effectiveness is traditionally estimated only through student attainment measures, such as the number or percentage of students who graduate (Grosskopf et al., 2014; Podinovski et al., 2014), standardized test scores in various subjects (Crespo-Cebada et al., 2014; Johnson and Ruggiero, 2014), scores on international and national assessments, or the percentage of students progressing to higher or further education (OECD, 2008c). However, it could be argued that the “results” of a school in terms of non-academic achievement should also be considered as educational objectives given that students with low levels of well-being are more likely to have a negative experience of school, as well as to suffer from depression and be involved in substance abuse or delinquency (Sun and Shek, 2010). As a result of the shared concerns of educational communities and families around the world, the latest trends aim to extend the focus of school effectiveness research beyond simple cognitive performance and also examine aspects such as well-being in the academic context. Some studies have focused on the effect of school on socio-emotional factors such as attitude to learning or academic self-concept (Opdenaker et al., 2002; Murillo and Hernández-Castilla, 2011; Belfi et al., 2012), although the results are not conclusive. Aware of the importance of socio-emotional development as an inseparable element of the integral learning process, the 2015 edition of the Programme for International Student Assessment PISA) incorporated a new instrument aimed at evaluating the socio-emotional variables related to the well-being of students, making it possible to assess school effectiveness in terms of improvements in student well-being at the international level.

### Well-Being

In recent years, the importance of well-being and the quality of life concept has grown and has extended into many areas. There are numerous definitions of these, and other terms such as satisfaction and happiness, that, as Veenhoven (2000) points out, have traditionally been used interchangeably. There is, however, nowadays consensus that quality of life refers to both objective and subjective elements and reflects both the living conditions and the perceptions of individuals (Casas, 2004). Moyano-Díaz and Ramos-Alvarado (2007) also assume an integrative perspective based on a model where the quality of life measure is divided into an objective component that refers to a person’s ability to access goods and services and a subjective one that incorporates the concept of subjective well-being, which, in turn, is divided into a cognitive and an affective component. In this case, the cognitive focuses on satisfaction (both global and in terms of specific domains), while the affective includes both positive and negative affects.

Assessing the impact of well-being on academic performance has also been the objective of several studies, the results of which have been equally diverse. For example, Novello et al. (1992) proposed a possible relationship between health and performance in which well-being seemed to play an important role, and, in the same vein, Berger et al. (2011) found, through a multilevel analysis, a relationship between socio-emotional well-being, well-being, self-esteem, social integration, positive perception of a school’s ambience, and performance. Gutman and Vorhaus (2012) also found, in a longitudinal study, significant correlations between four dimensions of well-being (emotional, behavioral, social, and school) and performance. However, El Ansari and Stock (2010) found that the relationship between health, educational performance, and well-being, the latter operationalized in terms of motivation and satisfaction with the educational experience, was reciprocal.

However, the concept of well-being in childhood and adolescence in itself has been studied extensively (Casas, 2010). For instance, Pollard and Lee (2003) carried out a systematic review where they found that, although well-being has not been defined consistently and there is no agreement on the best way to measure it, five key dimensions are usually addressed (albeit not usually all at the same time), namely, physical, psychological, cognitive, social, and economic well-being. The physical dimension refers to health and physical habits; the psychological to emotions and mental health (often operationalized by the “absence” of negative indicators); the cognitive to intellectual and school-related elements; the social to relationships with others, support, and interpersonal or communicative skills; and the economic to economic resources of the family.

An international survey, PISA, in its addition of 2015, adopted a comprehensive model in the evaluation of well-being, which incorporates, in addition to the habitual evaluation of performance, items and scales aimed at measuring well-being. The PISA 2015 assessment formulates a model including indicators of five dimensions of well-being: psychological, social, physical, material, and cognitive (Borgonovi and Pál, 2016). The model differs from the proposals described above by incorporating in the material dimension aspects related to educational and cultural resources.

In the present study, we use the definition of well-being from the PISA theoretical framework, which describes it as “*a dynamic state characterised by students experiencing the ability and opportunity to fulfil their personal and social goals. It encompasses multiple dimensions of students’ lives, including: cognitive, psychological, physical, social and material. It can be measured through subjective and objective indicators of competencies, perceptions, expectations and life conditions*” (Borgonovi and Pál, 2016).

Furthermore, the OECD has published recently the unified framework for the assessment of social and emotional skills (Kankaraš and Suárez-Álvarez, 2019), one of the fundamental pillars of well-being, which reiterates the importance of socio-emotional development of individuals, crucial for students’ maturity. The OECD defines these skills as “…individual capacities that can be (a) manifested as consistent patterns of thoughts, feelings and behaviors, (b) developed through formal and informal learning experiences, and (c) important drivers of socioeconomic outcomes throughout the individual’s life” (OECD, 2015).

### School Effectiveness

School effectiveness has been examined in hundreds of studies since the publication of the Coleman Report in 1966 (Coleman et al., 1966). The conclusions of this report highlighted the low impact of school factors on student performance in comparison with the strong effect exerted by family socioeconomic context, which educational institutions were ill-equipped to counter. That said, Coleman did also offer the first estimations of school effects, finding that the educational institution explains from 5 to 9% of the variance in mathematics results. Since then, a significant amount of work has been carried out that aims to identify the various factors related to performance and to quantify the magnitude of school effects on students’ results (MacBeath and Mortimore, 2001; Hanushek and Luque, 2003; Scheerens and Demeuse, 2005). Teddlie et al. (2000), in the *International Handbook of School Effectiveness Research*, summarized the most important evidence in this field, concluding that there is great variation in estimates of school effectiveness between countries and depending on the methodological approach taken. In general, 5–35% of the variance in academic achievement results between schools is explained by educational policies and practices, a school’s atmosphere, and learning climate, depending on the study involved (Martínez-Arias, 2009).

Studies that focus on school effectiveness in terms of the promotion of non-cognitive variables are much less common, although there are some notable exceptions. Murillo and Hernández-Castilla, 2011 performed a cross-country study in Latin America and Spain to estimate the magnitude of school, classroom, and country effects for non-cognitive variables such as self-concept, classroom behavior, social coexistence, and students’ satisfaction with their school. Belfi et al. (2012) conducted a literature review of the influence of class composition (ability and gender) in secondary education on students’ school well-being and academic self-concept. Lazarides and Buchholz (2019) studied the relationship between student-perceived teaching quality in mathematics classrooms and enjoyment, anxiety, and boredom, at both student and classroom levels, and estimated that these parameters accounted for 4–10% of school effects depending on the variable. Other studies in this area include those by Grisay (1996); Opdenakker and Van Damme (2000), Opdenaker et al. (2002); Sammons (1999), and Vandenberghe et al. (1994), all of which report schools’ minimal impact on non-cognitive educational results and attribute less than 5% of variation to the educational institution.

The present study has two main objectives. The first is to identify the well-being components that significantly affect student academic performance. The second consists in estimating the magnitude of school effects on the well-being of students in the OECD countries, where school effect is understood as the ability of schools to increase students’ subjective evaluation of their well-being. In addition, the relationship between socio-emotional variables and student- and school-level factors is examined.

## Method

### Sample

The PISA database developed by the OECD is the main source of information used in this study. PISA aims to evaluate the knowledge and skills acquired by students at the end of compulsory education in OECD member countries (35 countries at the time of the 2015 PISA report) and in non-member countries that have joined the project. The test systematically evaluates three areas of knowledge, reading, mathematics, and science. PISA evaluations are organized in such a way that in each cycle (PISA evaluations are carried out every 3 years), one of the evaluation areas is examined in depth. PISA 2015, the sixth edition of the study, focused on science achievement. In the present study, the full data set from all the OECD countries has been used, which comprises data collected from 248,620 15-year-old students. The summed data of all OECD countries were used to obtain the total OECD results, and the individual country data sets were employed for cross-country analysis. Table 1 reflects sample configuration by country (sample size and percentage of girls), along with the country abbreviation used throughout the study.

**TABLE 1:** Sample configuration.

Abbreviation	Country	Total	% of girls
AUS	Australia	14,530	49
AUT	Austria	7,007	49
BEL	Belgium	9,651	49
CAN	Canada	20,058	50
CHL	Chile	7,053	50
CZE	Czech Republic	6,894	50
DNK	Denmark	7,161	50
EST	Estonia	5,587	50
FIN	Finland	5,882	49
FRA	France	6,108	51
DEU	Germany	6,504	49
GRC	Greece	5,532	49
HUN	Hungary	5,658	50
ISL	Iceland	3,371	52
IRL	Ireland	5,741	49
ISR	Israel	6,598	56
ITA	Italy	11,583	50
JPN	Japan	6,647	50
KOR	Korea	5,581	48
LVA	Latvia	4,869	50
LUX	Luxembourg	5,299	51
MEX	Mexico	7,568	50
NLD	Netherlands	5,385	50
NZL	New Zealand	4,520	50
NOR	Norway	5,456	50
POL	Poland	4,478	49
PRT	Portugal	7,325	50
SVK	Slovak Republic	6,350	48
SVN	Slovenia	6,406	45
ESP	Spain	6,736	51
SWE	Sweden	5,458	50
CHE	Switzerland	5,860	48
TUR	Turkey	5,895	50
GBR	United Kingdom	14,157	49
USA	United States	5,712	50
OECD total		248,620	50

### Instruments

The cognitive test in PISA 2015 aimed to evaluate the level of acquisition of competences in science, reading, and mathematics, and the student questionnaire collected information about the students themselves, their family background, and school and learning environment. Additionally, school principals completed a questionnaire about the school, its resources, and management practices, and in some countries, optional teacher and parent questionnaires were also used. In this study, only the data relating to the student and school questionnaires as well as the performance test results were analyzed since the teacher and parent data are not available for many OECD countries.

The cognitive performance scale in PISA has become a worldwide reference as it is based on internationally agreed-upon theoretical frameworks. PISA uses the concept of competences, which in this context refers to the ability of students to extrapolate what they have learned and apply their knowledge and skills in real-life situations, as well as their ability to analyze, reason, and effectively communicate their findings and interpret and solve problems in different situations. The full PISA cognitive performance test comprises 528 questions about science, mathematics, reading, problem solving in collaboration, and financial competence and in total constitutes 13 h of tests. However, the test is constructed using a matrix design such that each student only answers a specific and limited combination of questions, resulting in a test that lasts approximately 2 h. Since the PISA 2015 edition focused on science, this field of study was evaluated in greater detail, and hence, the number of items evaluating this area was higher than for other areas, a total of 184 items, which equates to about 6 h in terms of test time, although each student only answers a (different) subset of these questions (for more details on the design, see the PISA 2015 Theoretical Framework: OECD, 2016a).

The student questionnaire collected demographic data of the students and their perceptions of their school environment, their learning experience, the processes and practices employed by the school, and students’ behavior. Based on students’ self-reports, a number of instruments were constructed: simple indexes (i.e., gender, age, or repetition of the same school grade) and complex indexes (economic, social, and cultural status (ESCS), an index of the disciplinary climate in the classroom, index of instrumental motivation, etc.).

In terms of the new element added to the PISA study in 2015, that is, the assessment of both subjective and objective measures of student well-being, as mentioned earlier, five dimensions were examined in order to consider well-being as a multidimensional element.

The cognitive dimension comprises students’ self-beliefs about their acquisition of subject-specific skills. As science was the main domain in PISA 2015, the questions regarding self-beliefs related to this area of knowledge. The constructs measured were: *science self-efficacy*, *broad interest in science*, *interest in broad science topics*, and *instrumental motivation to learn science*.

The psychological dimension encompassed psychological functioning in relation to educational aspects such as *students’ career and educational expectations*, measured in terms of the *expected job* and *the highest level of education* each student aspired to, *achievement motivation*, and *test and learning anxiety*, along with the *overall satisfaction with life*.

The physical dimension in PISA 2015 measured two aspects of students’ lifestyle: *the amount and frequency of physical activity* and *eating habits*. Specifically, students were asked if they exercised or did any sport before or after going to school, how many days per week they had physical education classes in school, and how often they were engaged in moderate or vigorous physical activities outside school. Students also reported whether they ate breakfast before going to school and dinner in the evening after school.

The assessment of the social well-being dimension was particularly important, as the quality of 15-years old relationships with teachers and peers is strongly linked to subjective well-being perception. PISA 2015 assessed five aspects of social well-being: *students’ sense of belonging at school*; *social learning experiences*, assessed through the value given to and enjoyment of cooperative learning; *the relationship with their teachers*, assessed through the perception of teachers’ unfair treatment of students; *the relationship with their peers*, as measured by the constructs *engagement with peers* and *bullying*; and *the relationship with their parents*, assessed through the scales *parental support* and *engagement with parents*.

Lastly, the material dimension investigated both the material resources available in the students’ households and the infrastructure of their school. The material conditions at home focused on *parental occupation status* and *physical resources at home*, data that also contributed to the computation of ESCS. Moreover, the students were asked if they *worked for pay* or *worked in households* before or after school. Information about the quality of the material environment of the school was collected through the questionnaire directed at school principals, which sought to quantify *human resources* in terms of the professional profile of the teachers employed by the school and any staff shortages, *material resources*, measured as the availability of *physical educational resources* and *computer availability*, and lastly, the *extracurricular activities* offered by the school. Table 2 describes the well-being model based on the OECD well-being framework.

**TABLE 2:** Well-being model dimensions.

Dimension	Constructs
Cognitive dimension	Enjoyment of science
	Instrumental motivation in science
	Science self-efficacy
	Interest in broad science topics
Material dimension	Parental occupation
	Physical resources at home
	Shortage of educational material
	Shortage of educational staff
	Index proportion of all teachers fully certified
	Total number of all teachers at school
Physical dimension	Eating breakfast/dinner
	Exercise or practice sport outside of school
Psychological dimension	Overall life satisfaction
	Achievement motivation
	Students’ career and educational expectations
	Test and learning anxiety
Social dimension	Belongingness at school
	Relationship with teachers: teacher fairness
	Collaboration and teamwork dispositions: enjoy cooperation
	Collaboration and teamwork dispositions: value cooperation
	Bullying

The original version of the student questionnaire can be found in Annex A of the PISA 2015 Theoretical Framework (OECD, 2016a), while the items of the specific well-being scales and constructs are collated in “A Framework for the Analysis of Student Well-Being in the PISA 2015 Study” (Borgonovi and Pál, 2016).

### Procedure

The students participating in PISA 2015 took a computer-based test, with assessments lasting a total of 2 h for each student. They also answered a background questionnaire, which took around 35 min to complete. The data collected were processed and published by OECD.

To achieve the objectives of our study, we used OECD data to perform a two-step analysis. Firstly, the well-being model configured through the dimensions or components that significantly impact students’ performance in an international context was identified. As a preliminary step, each dimension of the proposed model was analyzed individually, discarding variables until the model adequately fitted the data. Then, the well-being-performance model was constructed by introducing science performance (the major domain of the 2015 edition of PISA) as the dependent variable. Science performance was estimated as the mean of the 10 plausible values, the estimators of student proficiency used in PISA. The proposed well-being model was configured for the whole sample of the OECD students.

Secondly, the magnitude of school effects in terms of the various measures of well-being were estimated at the international and country level. With this purpose, the gross variance of the well-being indicators accounted for by clustering as well as the variance adjusted by students’ characteristics were assessed.

In addition, the relationships between student/school-level factors and the well-being indicators at the international and country level were analyzed. With this purpose, the previous model was enriched with the predictor variables related to school characteristics.

### Data Analyses

During the first step, the well-being model was evaluated using confirmatory factor analysis (CFA), where the latent variables were those represented by student responses to the student questionnaire. The estimation method employed was maximum likelihood with robust standard errors. The fit of the model was analyzed according to different criteria: the comparative fit index (CFI), the Tucker–Lewis index (TLI), the root mean square error of approximation (RMSEA), and the standardized root mean square residual (SRMR), taking into account the usual criteria as set out in Hu and Bentler (1999): CFI and TLI should be greater than 0.95, RMSEA should be below 0.06, and SRMR below 0.08. Then, the multiple regressions for the OECD countries as a whole and for individual countries were used to compute the standardized beta weights and the percentage of variance in academic achievement as a function of the studied variables. CFA was carried out using the *lavaan* package of R software (Rosseel, 2012), and multiple regressions using the *rms* package (Harrell, 2019).

The second step aimed to measure, at the OECD level and the individual country level, school effectiveness in the promotion of the well-being dimension, as well as those variables identified in step 1 as being important in relation to performance. At the country level, the PISA data have a hierarchical structure, where the individuals at level 1 (students) are nested in clusters at level 2 (schools). It is generally accepted that school effectiveness studies require multilevel techniques, such as those developed by Aitkin and Longford (1986), to be employed both in order to estimate the magnitude of school effects and to analyze the impact of student- and school-related factors (Aitkin and Longford, 1986; Hill and Rowe, 1996; Kennedy and Mandeville, 2000; Goldstein, 2003; Murillo, 2008; Gamazo et al., 2018). In this work, therefore, hierarchical linear modeling was used to estimate school effects on well-being indicators (Snijders and Bosker, 2012) whereby the two-level technique was applied in the cross-country analysis, the first level corresponding to students and the second to schools.

The estimation of the variance components of the model allows the calculation of the intraclass correlation coefficient (ICC), which represents the proportion of variation in dependent variables that is accounted for by clustering (Snijders and Bosker, 2012), i.e., ICC, is the ratio of the between-school variance to the sum of the between-school and within-school variance. ICC was calculated in two phases.

#### Phase 1. Null Model Estimation

In the first phase, gross school effects were estimated through the null model, which contained only the dependent variables and the constant. In this configuration, the model has random effects at both levels without taking into account any control variables. The null model is usually established as the starting point of multilevel analysis. It makes it possible to obtain the “gross” school effects, assessed through the ICC, i.e., those effects that are not adjusted for contextual variables (Lee, 2000; Hayes, 2006).

#### Phase 2. Estimation of the Model Incorporating Adjustment Variables

There is a consensus that school effects cannot be measured in terms of “gross” results but should be adjusted by relevant factors related to student progress (Goldstein et al., 1993; Mortimore et al., 1994; Goldstein and Thomas, 1996; Gray et al., 1996). To this end, in the second phase, the model was enriched with the control variables (Table 3), and the adjusted school effects measured in terms of ICC were estimated.

**TABLE 3:** Control variables.

Level	Variable
Student level	Economic, social, and cultural status
	Gender
	Immigration status
	Grade
School level	School-level economic, social, and cultural status

The ESCS index at the student level and the mean ESCS at the school level were incorporated in the model. These indicators have continuously been demonstrated to be strong predictors of school outcomes in all OECD countries (Perry and McConney, 2010a, b; Cordero et al., 2014; Suárez-Álvarez et al., 2014; OECD, 2016b; Gamazo et al., 2018). In PISA, the ESCS index is constructed from three components: *the occupational status of the parents*, *the educational level of the parents* (selecting in both cases the data for the parent with the higher level), and *home possessions*.

The impact of student-level background information, like gender and immigration status, has also been widely studied, the results underlining the importance of gender as a predictor of achievement (Stoet and Geary, 2013; Karakolidis et al., 2016; Özdemir, 2016). The model used in this work also included information about repetition of the same grade. Although its benefits are not compared between OECD countries here (Jacob and Lefgren, 2004, 2009; Manacorda, 2012), this strategy is widely used in some countries, like Spain and Portugal. For categorical variables like gender and immigrant status, dummy variables were generated (as many as the number of categories of the original variable minus one).

At the last stage of the study, with the purpose of assessing the relationship between student and school factors related to well-being, the complete model was configured whereby the predictor variables from the previous step were widened to include school factors such as school type, class size, or teaching methodology. There is evidence that supports the notion that these factors influence educational outcomes. For instance, the meta-analysis by Hattie (2009) suggests that reduced class size is a determining factor for improving student achievement, along with a reduced teacher–student ratio (Nath, 2012).

The model was also enriched with the variables that evaluated teaching strategies and teacher support, concepts that have recently gained interest in the academic field (Hattie, 2009; Nath, 2012; Gil et al., 2018) with respect to measuring their effects on student well-being. The OECD classifications distinguish between teacher-directed and student-centered instruction methodologies. Teacher-directed instruction, assessed through the scale *teacher-directed science instruction*, is focused on the role of teacher leading and managing the activities taking place in the classroom. Student-centered instruction, referred to as *inquiry-based science teaching and learning practices*, is associated with the teacher facilitating students’ own learning by allowing them time to find solutions to problems on their own before the teacher confirms or demonstrates the solution (Hoad et al., 2007; Rowe, 2007; OECD, 2009).

*Teacher support* is also gaining importance (OECD, 2016b; Ricard and Pelletier, 2016). Following the PISA measurement construct, teacher support consists in the teacher showing an interest in every student’s learning separately, giving extra help when needed, helping students with their learning, continuing to work on a teaching point until all students understand the material, and giving students an opportunity to express their opinions.

The school-level variables, i.e., teaching methodology and teacher support, were calculated as the across-school average of these student-level indexes, constructed on the basis of student responses to the context questionnaires following PISA methodology (OECD, 2017b). The predictor variables of the complete model are shown in the Table 4. The package lme4 of R software was used for multilevel modeling (Bates et al., 2015).

**TABLE 4:** Predictor variables.

Level	Variable
Student level	Economic, social, and cultural status
	Gender
	Immigration status
	Grade
School level	School type
	Student–teacher ratio
	School size
	Class size
	Teacher-directed science instruction (school level)
	Inquiry-based science teaching and learning practices (school level)
	Teacher support of students’ choices in a science classes (school level)

## Results

### Relationship Between Student Performance and Well-Being

As a preliminary step to data analysis, for each dimension of the proposed well-being model, a CFA was performed on the summed data for all OECD countries. The cognitive dimension, represented by the four constructs explained above, was the only one that achieved appropriate model fit according to the criteria of Hu and Bentler (1999). The material dimension, defined as the economic resources of a student’s household, represented through the *home possessions* index and the index of parental occupational, was also confirmed. Although psychological, social, and physical dimensions, assessed through the respective OECD scales, did not exhibit construct solidity, individual scales aimed at assessing these dimensions were introduced into the well-being-performance model in order to capture whether, and how, subjective non-cognitive well-being indicators predict performance in science.

**FIGURE 1:**
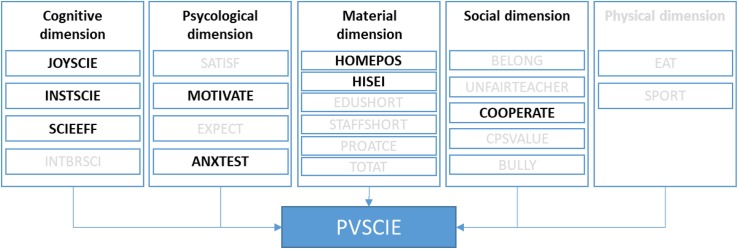
Confirmed well-being/performance model. Source, Prepared by the authors, based on Programme for International Student Assessment (PISA) Well-being Framework.

Figure 1 presents the final well-being-performance model, which is the one that achieved the highest fit values. Table 5 indicates the values obtained for the CFA. In the model representing the relationship between well-being and performance, the cognitive dimension was finally reduced to three scales: *enjoyment of science*, *instrumental motivation in science*, and *science self-efficacy*. In the psychological dimension, the variables *achievement motivation* and *test anxiety*, considered individually, acted as good predictors of science performance. The material dimension, measured through the level of *parents’ occupation* and *home possessions* of students’ families, was strongly related to performance. Finally, although four social dimension variables (*belongingness at school, teacher fairness, enjoy cooperation*, and *value cooperation)* were demonstrated to have a significant impact on science achievement, the model that included all four of them did not fit the data well. However, the variable *enjoy cooperation* contributed positively to the final model. The variables of the physical dimension did not provide reliable information about the well-being-performance model, probably due to their dichotomous nature.

**TABLE 5:** Model fit test statistics of well-being-performance model.

	χ^2^	CFI	TLI	RMSEA	SRMR
Total OECD	144701.688***	0.957	0.953	0.038	0.036

*^∗∗∗^Significant at *p* < 0.01; CFI, comparative fit index (CFI); TLI, Tucker–Lewis index; RMSEA, root mean square error of approximation; SRMR, standardized root mean square residual.*

Multiple regression (Table 6) indicated that well-being variables explained around 22% of the variance related to students’ achievement in science.

**TABLE 6:** Regression coefficients.

Abbreviations	Country	*R*^2^	Beta				
			
			COGWB	MATWB	MOTIVATE	ANXTEST	COOPERATE
Total OECD		0.224	17.88***	31.44***	0.41***	−12.24***	4.35***
AUS	Australia	0.238	28.73***	25.69***	5.25***	−8.85***	2.00***
AUT	Austria	0.247	16.41***	34.56***	2.28***	−16.02***	1.74***
BEL	Belgium	0.256	20.07***	38.00***	−5.73***	−10.12***	7.98***
CAN	Canada	0.197	21.77***	21.22***	5.15***	−11.67***	1.39***
CHL	Chile	0.241	4.93***	29.52***	5.95***	−18.01***	8.50***
CZE	Czech Republic	0.317	14.69***	42.68***	6.47***	−15.94***	8.46***
DNK	Denmark	0.280	21.76***	26.05***	12.41***	−14.40***	1.83***
EST	Estonia	0.273	16.64***	22.74***	9.65***	−17.89***	5.65***
FIN	Finland	0.262	24.39***	23.68***	11.72***	−20.38***	NS
FRA	France	0.279	22.00***	36.99***	NS	−10.44***	9.44***
DEU	Germany	0.184	18.03***	39.22***	1.14***	−12.93***	6.59***
GRC	Greece	0.247	18.66***	27.13***	9.35***	−11.37***	5.10***
HUN	Hungary	0.295	3.26***	43.61***	7.54***	−12.87***	10.52***
ISL	Iceland	0.229	17.16***	14.48***	12.56***	−16.23***	2.30*
IRL	Ireland	0.220	27.45***	23.42***	7.55***	−13.31***	−1.19***
ISR	Israel	0.258	13.11***	29.54***	2.64***	−6.88***	−0.50*
ITA	Italy	0.277	15.98***	30.97***	−1.58***	−9.20***	9.08***
JPN	Japan	0.214	23.89***	24.37***	5.71***	−1.80***	−0.88***
KOR	Korea	0.130	25.08***	25.06***	11.60***	1.12***	−1.35***
LVA	Latvia	0.180	10.80***	23.41***	11.15***	−18.23***	12.67***
LUX	Luxembourg	0.168	17.35***	39.38***	NS	−16.33***	6.02***
MEX	Mexico	0.209	4.68***	17.17***	8.64***	−12.43***	5.16***
NLD	Netherlands	0.341	23.29***	36.80***	6.69***	−1.71***	4.89***
NZL	New Zealand	0.192	27.67***	29.33***	3.61***	−15.71***	1.71***
NOR	Norway	0.173	24.57***	24.94***	9.03***	−11.76***	3.42***
POL	Poland	0.198	12.96***	28.40***	9.91***	−15.64***	7.03***
PRT	Portugal	0.228	18.02***	30.10***	13.50***	−15.43***	−1.87***
SVK	Slovak Republic	0.247	11.03***	34.18***	11.21***	−9.54***	14.63***
SVN	Slovenia	0.197	17.10***	33.63***	6.97***	−12.89***	10.96***
ESP	Spain	0.287	20.62***	24.50***	10.44***	−16.14***	5.24***
SWE	Sweden	0.224	22.96***	29.19***	6.33***	−9.23***	2.95***
CHE	Switzerland	0.201	18.54***	39.44***	2.06***	−13.14***	4.49***
TUR	Turkey	0.226	10.87***	26.58***	4.36***	−5.14***	6.19***
GBR	United Kingdom	0.188	27.71***	28.21***	−0.37***	−6.30***	1.99***
USA	United States	0.182	18.35***	26.93***	0.26***	−10.71***	2.21***

***Significant at p < 0.01; *significant at p < 0.1; NS, not significant. COGWB, cognitive well-being; MATWB, material well-being; MOTIVATE, achievement motivation; ANXTEST, test and learning anxiety; COOPERATE, enjoy cooperation.

It can be observed that in the regression model performed for the overall OECD sample, the greatest weight corresponded to the material well-being dimension. Nevertheless, the impact of cognitive well-being is also both high and constant across countries: on average, an increase of 1 point in terms of cognitive well-being would result in an increment of 18 points on the PISA science achievement scale. In six countries (Australia, Canada, Finland, Iceland, Ireland, and Korea), the cognitive variables are able to predict achievement as much as, or in some cases better than, economic background does. Test anxiety was found to reduce science performance by up to 12 points, with the strongest negative relationship observed in Finland. These results are also constant across countries, excepting Korea, where higher test anxiety corresponds to higher performance in science. *Achievement motivation* and *enjoy cooperation* both also relate positively to the cognitive results in most of the countries, although their impact is weaker.

### School Effectiveness in Well-Being Promotion

Once the well-being components that were strongly related to performance were identified, we studied the school effects on the well-being components that can be modulated by the school. These effects on science cognitive scores are also presented.

Table 7 summarizes the school effects for the null model and for the model adjusted for student background and ESCS information. Consistent with previous research, the results indicate that the school seems to have only a weak influence on student well-being, although there is some variation depending on the country analyzed and on the predictor variable considered. In the null model, the total OECD school-level variation in science performance was around 39%, while it barely reached 9% for the well-being components, indicating that the school’s role turns out to be much less important in promoting students’ well-being. School effects accounted for 9% of variation in the cognitive well-being dimension, 8% of test anxiety, and 5% of enjoyment of cooperation. The model adjusted to incorporate the control variables did not result in any significant differences in terms of school effects, explaining only 1% of variation for enjoyment of science and enjoyment of cooperation variables, while the school-level variation in science performance was reduced up to 25%.

**TABLE 7:** School effects in terms of ICC.

	SCIE	COGWB	JOYSCIE	SCIEEFF	INSTSCIE	MOTIVATE	ANXTEST	COOPERATE
**Gross school effect**								
Total OECD school effect	39.0%	9.0%	9.0%	5.0%	7.0%	3.0%	8.0%	5.0%
Min school effect	5.0%	2.2%	2.1%	1.7%	0.5%	0.5%	0.6%	1.0%
Max school effect	62.1%	13.7%	10.7%	7.2%	12.6%	9.3%	10.0%	6.3%
**Net school effect**								
Total OECD school effect	25.0%	9.0%	8.0%	5.0%	7.0%	3.0%	8.0%	4.0%
Min school effect	3.6%	0.8%	0.9%	0.4%	0.3%	0.3%	0.4%	0.3%
Max school effect	41.5%	10.8%	8.6%	3.9%	11.8%	8.7%	10.3%	6.0%

*ICC, intraclass correlation coefficient; SCIE, science performance; COGWB, cognitive well-being; JOYSCIE, enjoyment of science; SCIEEFF, science self-efficacy; INSTSCIE, instrumental motivation in science; MOTIVATE, achievement motivation; ANXTEST, test and learning anxiety; COOPERATE, enjoy cooperation.*

**FIGURE 2:**
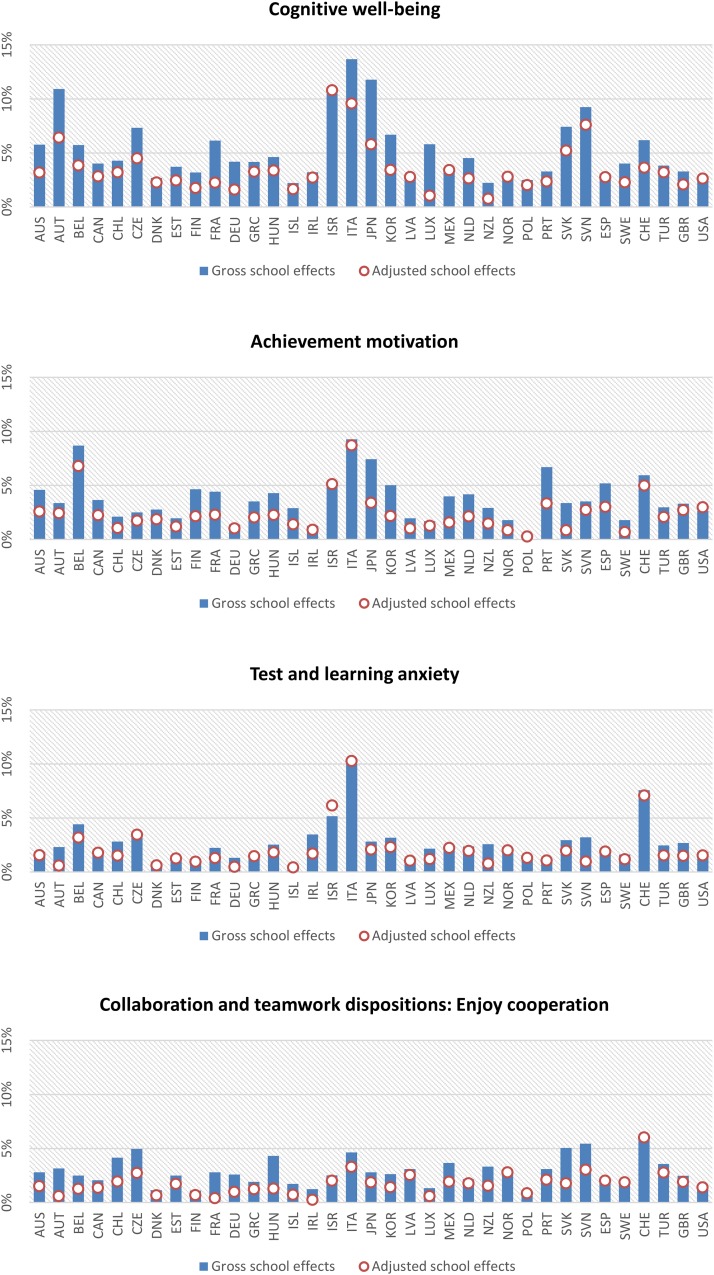
Country-level school effects in terms of intraclass correlation coefficient (ICC).

Figure 2 reflects cross-country school effects for the cognitive dimension and for the psychological and social variables (for the country-level results and for the variables that compose the cognitive well-being dimension, please refer to Supplementary Material). In comparison with the rest of the variables, the role of the school in cognitive well-being promotion is systematically higher than other dimensions in OECD countries. Adjusted school effects in Italy and Japan were around 10% in terms of cognitive well-being. Italy, along with Belgium, also showed higher variability in students’ perception of achievement motivation at the school level. Enjoyment of cooperation is the component of social well-being where schools had less impact, a result that is consistent across countries, with Switzerland being the only country where it exceeded 5%. Schools also do not seem to play any great part in test anxiety reduction. Italy was the only country where any great amount (10%) of variation in this dependent variable was accounted for by school nesting. In Iceland and Germany, no school variation in test anxiety was observed.

### Student and School Factors Related to Performance

Finally, the impact of student and school factors on well-being variables was assessed. The results for science performance are presented in order to reflect the differences in the influence of these factors on achievement results and well-being. Table 8 shows the estimates of multilevel modeling on each dependent variable for the overall OECD sample. Table 9 identifies the number of countries where the factors are significantly positively related to the dependent variables.

**TABLE 8:** Estimation of fixed effects and random effects of the complete model for the overall OECD sample.

	PVSCIE	COGWB	JOYSCIE	SCIEEFF	INSTSCIE	MOTIVATE	ANXTEST	COOPERATE
**Student level**								
ESCS	19.65***	0.17***	0.15***	0.22***	0.08***	0.14***	−0.06***	0.09***
GENDER_girl	−8.08***	−0.13***	−0.13***	−0.20***	−0.02***	0.02***	0.45***	0.21***
IMMIG_yes	−18.29***	0.10***	0.13***	0.03**	0.09***	0.19***	0.08***	0.06***
**School level**								
SCHLTYPE_pub	−11.63***	0.04***	0.03***	0.06***	0.01	−0.08***	0.03***	−0.05***
STRATIO	0.00	0.00***	0.00**	0.00**	0.00***	0.00***	0.00	0.00***
SCHSIZE	0.00	0.00	0.00***	0.00***	0.00	0.00***	0.00	0.00***
CLSIZE	0.46***	0.01***	0.01***	0.01***	0.01***	0.01***	0.01***	0.01***
TDTEACH_S	52.59***	0.18***	0.29***	0.06***	0.10***	0.05**	0.04***	0.17***
IBTEACH_S	−27.20***	0.17***	0.13***	0.24***	0.07***	0.09***	−0.07***	0.04***
TEACHSUP_S	−24.97***	0.23***	0.21***	0.09***	0.28***	0.33***	0.21***	0.05***
**Random effects**								
σ2	5248.41(71%)	0.88(95%)	1.15(96%)	1.44(97%)	0.94(96%)	0.81(87%)	0.87(94%)	0.94(97%)
τ00 (CNTSCHID)	2116.7(29%)	0.05(5%)	0.05(4%)	0.05(3%)	0.04(4%)	0.12(13%)	0.06(6%)	0.03(3%)

***Significant at p < 0.01; **significant at p < 0.05; NS, not significant. CNTSCHID, country school ID; PVSCIE, science performance; COGWB, cognitive well-being; JOYSCIE, enjoyment of science; SCIEEFF, science self-efficacy; INSTSCIE, instrumental motivation in science; MOTIVATE, achievement motivation; ANXTEST, test and learning anxiety; COOPERATE, enjoy cooperation; ESCS, economic, social, and cultural status; GENDER_girl, gender (the student is a girl); IMMIG_yes, (the student is an immigrant); SCHLTYPE_pub, school type (the school is public); SCHSIZE, school size; STRATIO, student–teacher ratio; CLSIZE, class size; TDTEACH_S, teacher-directed science instruction (school level); IBTEACH_S, inquiry-based science teaching and learning practices (school level); TEACHSUP_S, teacher support of students’ choices in science classes (school level).

**TABLE 9:** Cross-country summary of fixed effects (number of countries with significant and positive impact).

Variable	SIG	ESCS	GENDER_girl	IMMIG_yes	SCHLTYPE_pub	SCHSIZE	STRATIO	CLSIZE	TDTEACH_S	IBTEACH_S	TEACHSUP_S
COGWB	SIG (N°)	35	34	18	5	0	7	4	19	17	17
	POSITIVE (N°)	35	3	13	3	0	3	4	19	17	15
JOYSCIE	SIG (N°)	33	25	16	6	6	9	7	29	12	17
	POSITIVE (N°)	33	2	15	2	0	6	6	28	10	15
SCIEEFF	SIG (N°)	35	30	13	9	7	6	5	13	18	8
	POSITIVE (N°)	35	1	12	3	0	4	3	11	18	4
INSTSCIE	SIG (N°)	31	19	14	5	3	6	3	4	14	17
	POSITIVE (N°)	31	7	14	3	0	4	2	3	14	16
MOTIVATE	SIG (N°)	34	26	22	12	7	3	6	12	6	10
	POSITIVE (N°)	34	12	21	4	0	2	4	9	3	8
ANXTEST	SIG (N°)	28	35	15	6	5	4	7	4	7	10
	POSITIVE (N°)	0	35	13	4	0	1	5	3	2	3
COOPERATE	SIG (N°)	33	34	12	9	10	4	11	20	4	13
	POSITIVE (N°)	33	34	9	3	0	1	7	19	1	12

*SCIE, science performance; COGWB, cognitive well-being; JOYSCIE, enjoyment of science; SCIEEFF, science self-efficacy; INSTSCIE, instrumental motivation in science; MOTIVATE, achievement motivation; ANXTEST, test and learning anxiety; COOPERATE, enjoy cooperation; ESCS, economic, social, and cultural status; GENDER_girl, gender (the student is a girl); IMMIG_yes, (the student is an immigrant); SCHLTYPE_pub, school type (the school is public); SCHSIZE, school size; STRATIO, student–teacher ratio; CLSIZE, class size; TDTEACH_S, teacher-directed science instruction (school level); IBTEACH_S, inquiry-based science teaching and learning practices (school level); TEACHSUP_S, teacher support of students’ choices in science classes (school level).*

ESCS has traditionally been positively related to performance, a tendency that, in this work, persists when well-being variables are taken into account. Students with higher ESCS exhibited higher cognitive well-being, with the strongest impact being on their perception of self-efficiency in science. More advantaged students also had higher achievement motivation and were more resistant to stress as a result of exams. This relationship was reproduced at the individual country level.

At the OECD level, girls demonstrated lower levels of cognitive well-being along with higher levels of test anxiety, although they enjoyed cooperation more than their male classmates and had higher levels of achievement motivation. At the individual country level, these results were repeated, except for achievement motivation, where, in 9 countries, no clear relationship with gender was observed, while in 14 countries, boys were more highly motivated to achieve academically.

Once ESCS was controlled for, students with immigrant backgrounds reported higher motivation to achieve than non-immigrant students. They also demonstrated higher levels of cognitive well-being, especially for enjoyment of science and instrumental motivation. On the other hand, being an immigrant was associated with higher test anxiety.

At the school level, the influence of school characteristics, along with teaching methods and teacher support, on the students’ subjective well-being was assessed. Although public schools consistently performed worse than private schools even after controlling for ESCS, this tendency was reversed in terms of students’ perception of their cognitive well-being. In public schools, students tended to demonstrate higher levels of self-efficiency and science enjoyment. Nevertheless, they were less motivated to achieve and more prone to feeling anxious about exams. The school and class size seemed to have a very low impact on students’ perception of well-being both at the OECD and at the individual country level.

Teaching methodology, measured as the use of teacher-directed or inquiry-based instruction, and teacher support are strongly and positively related to the well-being indicators, while they have an opposite effect in relation to science performance: the more frequent use of inquiry-based teaching and higher teacher support are associated with a decrease in science performance of around 25 points on the PISA scale. However, more inquiry-based instruction, when students are given opportunities to explain their ideas, spend time in the laboratory doing practical experiments, or are required to discuss science questions, increases students’ perception of self-efficiency and promotes intrinsic motivation by increasing science enjoyment. Furthermore, it reduces exam anxiety and raises achievement motivation. The positive relationship between inquiry-based teaching and the cognitive well-being dimension is confirmed individually in 17 OECD countries.

Enjoyment for science is higher when the teacher regularly explains scientific ideas, a whole class discussion takes place with the teacher, and the teacher addresses students’ questions and practically explains an idea. Teacher-directed instruction also increases students’ positive predisposition toward cooperation. The positive impact of teacher-directed methodologies on cognitive well-being and cooperation is observed in 19 separate OECD countries.

Teacher support was the school-level variable that demonstrated the strongest relationship with student well-being in the model proposed. Showing an interest in every student’s learning, giving extra help when students need it, and continuing with explanations until all students understand the material turn out to be extremely important for the promotion of achievement motivation and for positive predisposition toward cooperation. In addition, these practices reduce test anxiety in 7 of the 10 OECD countries where teacher support is significant.

## Discussion

The aim of the study was twofold. On the one hand, the present study sought to reach a global definition of well-being across the countries assessed for the PISA 2015 report and to assess its relationship with performance. On the other, we focused on ascertaining the impact of school effects on student welfare and identifying those factors positively related to well-being in the educational context.

The results evidenced the complexity of the well-being concept and the need for further research on its definition. Of the four dimensions described in the original model, only the cognitive dimension was confirmed as having an impact across all countries in PISA 2015. In the evaluation of the material dimension, only student-level variables contributed positively to the model, while school environment and resources did not demonstrate any significant effect once the students’ economic background was taken into account. Psychological and social dimensions were found to be multifaceted concepts represented by a variety of individual variables but not confirmed as solid constructs. Finally, the physical dimension did not provide reliable information with respect to the construct definition.

Consequently, in the well-being-performance model, well-being was finally defined by the cognitive and material dimensions, along with the individual psychological and social variables *achievement motivation*, *test anxiety*, and *enjoyment of cooperation*, i.e., the variables that were found to be good predictors of performance in science. The results showed that student well-being significantly impacts student performance. Higher cognitive well-being is associated with better achievement results, increasing science performance by up to 22 points on the PISA scale. In six countries, the promotion of cognitive well-being was even demonstrated to counteract the effect of socioeconomic background. Lower *test anxiety* is also linked to better results, along with *enjoyment of cooperation*.

Nevertheless, currently, school interventions do not appear strong enough to make an impact on subjective well-being. School effects explain barely 5% of the variation in well-being perception within schools, and school-level variation is highest for the cognitive well-being dimension, accounting for up to 9% of school effects on average across all the OECD countries. These results are consistent with previous studies (Murillo and Hernández-Castilla, 2011; Lazarides and Buchholz, 2019) and provide further evidence in support of these effects both in the across-OECD context as well as for each member country. Our results highlight that some countries, like Italy and Switzerland, are more successful with school-level interventions, while others, like Poland and Iceland, have a very limited school-level influence on well-being. There may be multiple reasons for this low school-level impact on well-being, the most likely being a lack of socio-emotional education within schools, the low availability of tools and policies for well-being improvement, or the limited time dedicated to achievement in non-academic aspects of learning (Murillo and Hernández-Castilla, 2011), although it is becoming more common to introduce school practices aimed at the promotion of cognitive, social, and emotional well-being and stress reduction (Jennings et al., 2013; Schonert-Reichl et al., 2015). Research findings provide evidence to support the notion that the implementation of such methods improves attention deficits, reduces stress, and promotes self-regulation among adolescents (Albrecht et al., 2012; Carboni et al., 2013).

The student and school factors associated with higher levels of cognitive well-being, motivation, and cooperation were also assessed. At the student level, the socioeconomic background was again a good predictor of student well-being, which clearly makes it difficult for schools to combat its substantial influence. However, on the positive side, the analysis provides evidence that teachers employing a methodology that combines the traditional teacher-led approach with more innovative practices based on inquiry and teamwork seems to be a powerful tool for improving non-cognitive educational achievement. Science teaching and learning practices that include experimentation and critical thinking increase students’ self-efficacy in science and reduce test anxiety. These insights are especially important given that student-oriented teaching methods seem to be negatively linked to academic achievement (Gil et al., 2018). A classic teacher-directed approach, where the teacher leads class discussions and explains ideas, is associated with higher levels of science enjoyment and better predisposition toward cooperative working. These results support the idea of the importance of an adaptive pedagogy that brings together innovation and teacher-directed instruction, rather than teachers opting exclusively for either one of these approaches (Rowe, 2007; OECD, 2008b).

Teacher support of pupils at the school level was initially negatively related to science performance in the multilevel model proposed here. This was probably due to the fact that teachers at schools in disadvantaged areas report supporting students in their learning more frequently than teachers in schools in more advantaged areas, as is also the case for teachers in rural as opposed to urban schools (OECD, 2017a). Schools in disadvantaged and rural areas tend to perform worse in the PISA assessments, and therefore, their students are in greater need of teacher support. Nevertheless, in this study, teacher support turned out to be the strongest predictor of student well-being, i.e., when the teacher works to ensure the students’ complete understanding of the problem, provides extra help when it is required, and aims to integrate learning, students report higher subjective well-being. Previous research (Ahmed et al., 2014) has also shown that student-perceived teacher support is negatively related to student anxiety and boredom at the student level, and positively related to enjoyment and negatively related to anxiety at the classroom level (Lazarides and Buchholz, 2019).

In the 21st century, the era of knowledge and innovation, the school has gained great importance in the development and learning of individuals, as well as it having become an extraordinarily complex and multidisciplinary facility. On the one hand, the purpose of schools is to promote knowledge acquisition, but on the other, they must help children build confidence and develop a variety of learning strategies for the future (OECD, 2008a). This research aims to contribute to the growing concern about students’ quality of life and happiness and to emphasize the importance of a comprehensive approach to education where socio-emotional development is integrated in a schools’ day-to-day functioning.

The principal limitation of the study lies in the need for improvement in the instruments available for assessing well-being in an educational context. Although the OECD provides a solid framework for the measurement of well-being, some dimensions, like physical well-being, still need to include reliable and unidimensional scales. Moreover, it should be taken into account that instruments based on self-reporting will never achieve the same level of sensitivity in measuring latent constructs such as those involved in well-being as do academic achievement tests (Murillo and Hernández-Castilla, 2011).

The results of this research should be considered with cautions, as there is no evidence of causality for the relationships observed. The reciprocal relationship between well-being and performance should be taken into account. For instance, previous research has shown that higher levels of achievement are positively related to enjoyment (Ma, 1997) and reduce exam anxiety (Ma and Xu, 2004). In addition, the impact of student and family characteristics should not be forgotten, as they are connected to the achievement and behavior of students at school, as García-Crespo et al. (2019) indicate. Nevertheless, the conclusions regarding teaching methodology are more consistent, although it would be interesting to study the persistence of the positive impact of teachers’ interventions with respect to students with different academic profiles (low/average/high academic performance, etc.). Future research within our research team will focus on expanding on the results obtained in this work by extending the analysis to primary education data, where it is expected that school involvement in socio-emotional variables is more common and efficient.

## Data Availability Statement

This study was based on the public data available on the OECD web page: http://www.oecd.org/pisa/data/2015database/.

## Ethics Statement

The study did not require ethics approval. The data collection was performed on behalf of the OECD following the standards for the protection of privacy and the processing of personal data (http://www.oecd.org/internet/ieconomy/oecdguidelineson the prote ction of pri vac yan dtra nsbo rde rflo wsof personaldata.htm).

## Author Contributions

EG prepared the data set for analysis, conducted the analysis, and was involved in writing the manuscript. IB and JM supervised the analysis and participated in writing the manuscript.

## Conflict of Interest

The authors declare that the research was conducted in the absence of any commercial or financial relationships that could be construed as a potential conflict of interest.
