# Supplementary material for: How Schools Affect Student Well-Being: A Cross-Cultural Approach in 35 OECD Countries
Source: Front Psychol. 2020 Mar 25;11:431. doi: 10.3389/fpsyg.2020.00431 (PMC7109313; doi:10.3389/fpsyg.2020.00431)
Supplement: Supplementary file 1 [file Data_Sheet_1.PDF]

Supplementary material

Figure I. Country level school effects in terms of ICC for cognitive dimension components

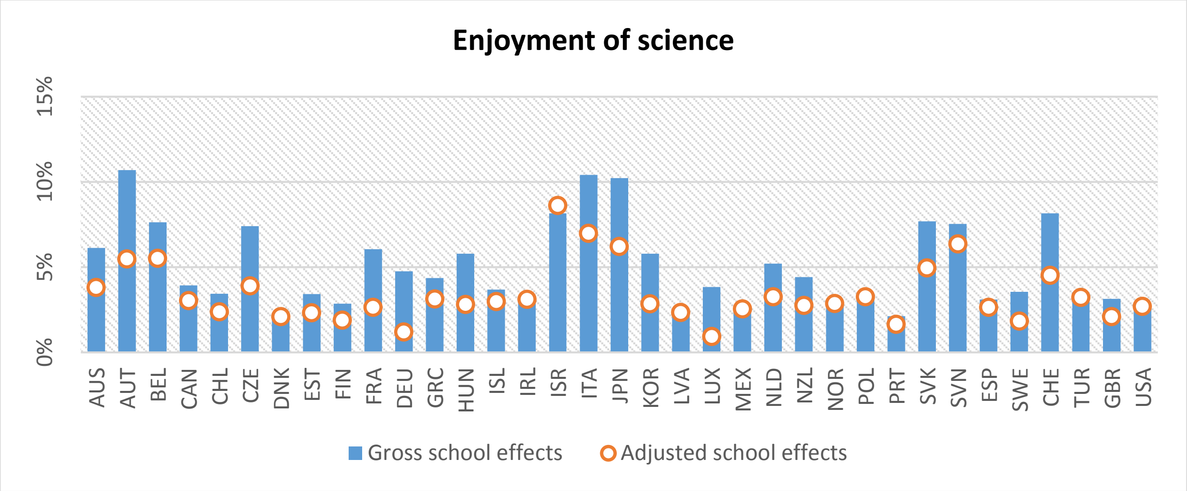

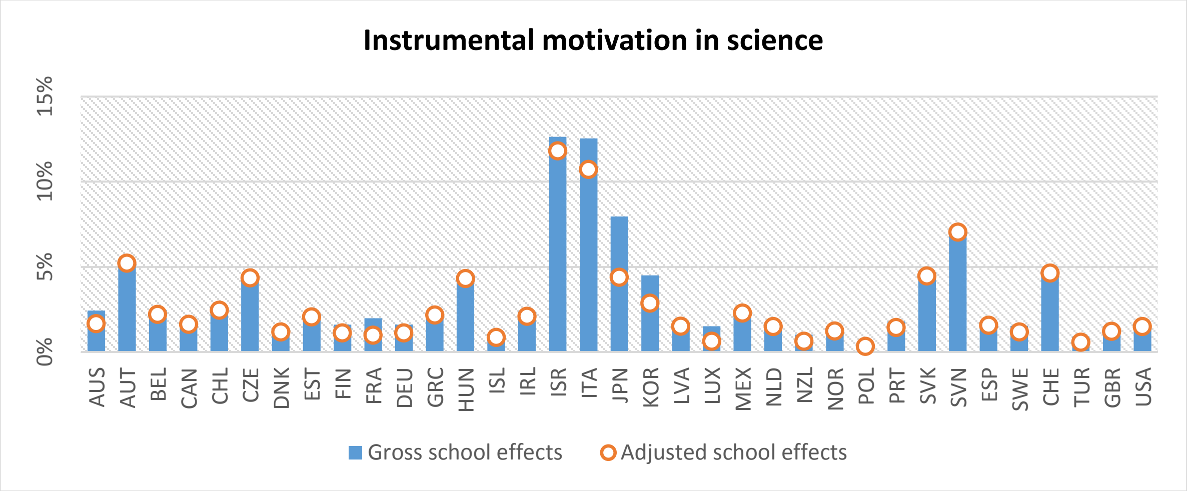

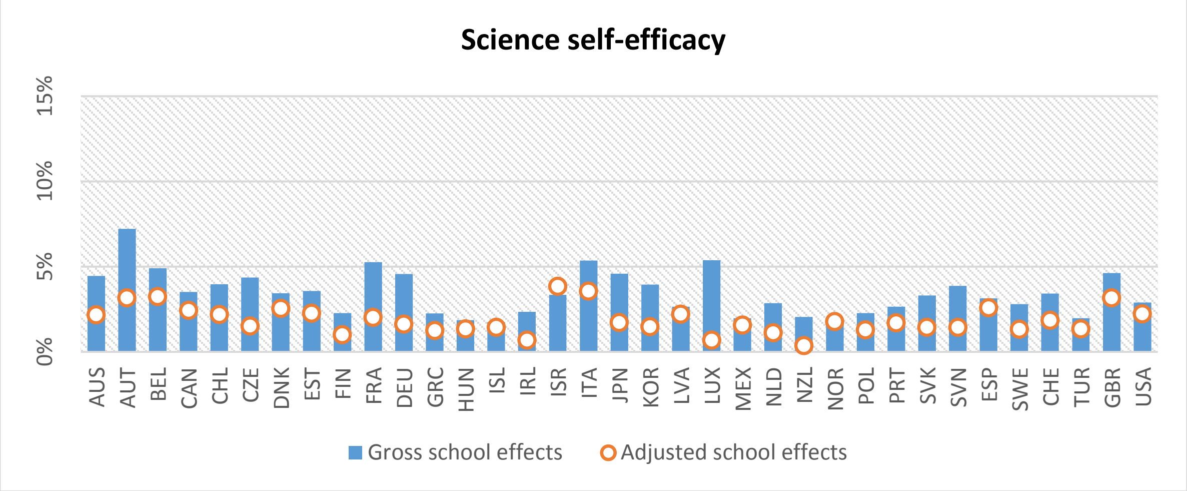

Table I. Cross-country estimation of fixed effects

COGWB									
**COUNTRY**	**ESCS**	**GENDER_girl**	**IMMIG_yes**	**SCHLTYPE**	**STRATIO**	**CLSIZE**	**IBTEACH_S**	**TDTEACH_S**	**TEACHSUP_S**
AUS	0.27***	-0.15***	0.22***	NS	NS	NS	0.13**	0.15***	0.21***
AUT	0.08***	-0.18***	-0.18***	NS	NS	NS	NS	NS	NS
BEL	0.2***	-0.22***	0.2***	NS	0.01**	NS	0.18**	0.33***	NS
CAN	0.27***	-0.09***	0.21***	0.08*	0*	NS	0.09*	NS	0.26***
CHE	0.18***	-0.18***	NS	NS	-0.02**	NS	NS	0.2***	NS
CHL	0.12***	0.06*	NS	0.11*	NS	NS	NS	NS	0.15*
CZE	0.15***	-0.06**	NS	NS	NS	0.01**	0.26***	0.13*	NS
DEU	0.14***	-0.41***	NS	NS	NS	NS	0.22*	0.28*	-0.21*
DNK	0.19***	-0.17***	0.26***	NS	NS	NS	0.2**	NS	0.18**
ESP	0.17***	-0.2***	0.16***	NS	NS	NS	NS	NS	0.16*
EST	0.18***	-0.06*	NS	NS	NS	NS	NS	0.26**	0.21***
FIN	0.27***	-0.1***	NS	NS	NS	NS	0.21***	0.33***	NS
FRA	0.25***	-0.34***	0.23***	NS	NS	0.02***	0.3***	0.22**	NS
GBR	0.22***	-0.19***	0.2***	-0.07*	0.02***	NS	NS	0.15*	0.19***
GRC	0.17***	-0.21***	NS	NS	NS	NS	0.15*	0.25***	NS
HUN	0.13***	-0.06*	NS	NS	NS	NS	0.24***	0.25***	NS
IRL	0.26***	-0.13***	0.13**	NS	NS	NS	NS	NS	0.2*
ISL	0.28***	-0.3***	NS	NS	NS	NS	NS	0.24*	NS
ISR	0.17***	-0.11***	0.12**	N/A	NS	NS	0.41***	NS	0.39***
ITA	0.15***	-0.18***	0.13***	0.12*	NS	NS	0.12*	0.44***	NS
JPN	0.21***	-0.39***	NS	NS	NS	NS	NS	0.37***	0.24**
KOR	0.3***	-0.23***	NS	NS	0.03*	NS	NS	NS	0.29**
LUX	0.15***	-0.15***	NS	NS	NS	NS	NS	0.52*	NS
LVA	0.13***	-0.07**	NS	NS	NS	NS	0.21**	NS	0.16**
MEX	0.02*	NS	-0.26**	NS	NS	NS	0.24***	NS	NS
NLD	0.15***	-0.27***	0.16*	NS	NS	NS	NS	0.33**	-0.31**
NOR	0.31***	-0.11***	0.26***	NS	NS	NS	NS	NS	0.15*
NZL	0.08***	-0.18***	-0.18***	NS	NS	NS	NS	NS	NS

POL	0.17***	0.08**	NS	NS	NS	0.01*	NS	0.19*	NS
PRT	0.14***	-0.13***	NS	NS	-0.01*	NS	0.21**	0.19*	NS
SVK	0.16***	-0.06*	NS	NS	NS	0.01*	0.31***	NS	NS
SVN	0.08***	-0.18***	-0.18***	NS	NS	NS	NS	NS	NS
SWE	0.08***	-0.18***	-0.18***	NS	NS	NS	NS	NS	NS
TUR	0.02*	0.09***	NS	NS	NS	NS	NS	0.39***	0.17*
USA	0.18***	-0.14***	0.16***	NS	-0.01*	NS	0.21***	NS	0.28***
**JOYSCIE**									
**COUNTRY**	**ESCS**	**GENDER_girl**	**IMMIG_yes**	**SCHLTYPE**	**STRATIO**	**CLSIZE**	**IBTEACH_S**	**TDTEACH_S**	**TEACHSUP_S**
AUS	0.21***	-0.15***	0.23***	NS	NS	NS	NS	0.23***	0.28***
AUT	0.24***	-0.22***	0.15***	NS	-0.01***	0.01*	0.1*	0.35***	NS
BEL	0.17***	-0.23***	0.18***	NS	0.02**	0.01*	NS	0.71***	NS
CAN	0.22***	-0.11***	0.23***	NS	NS	NS	NS	0.15***	0.31***
CHE	0.2***	-0.2***	NS	NS	-0.02**	NS	NS	0.26***	NS
CHL	0.1***	0.09**	NS	NS	0.01*	NS	NS	0.27**	0.19*
CZE	0.17***	NS	NS	NS	NS	0.01**	0.17**	0.31***	NS
DEU	0.19***	-0.45***	NS	NS	NS	NS	0.32**	0.34**	-0.27**
DNK	0.16***	-0.09**	0.22***	NS	NS	NS	NS	NS	0.18*
ESP	0.15***	-0.14***	0.23***	NS	NS	NS	NS	NS	0.23**
EST	0.16***	-0.07*	NS	NS	NS	NS	-0.18*	0.31**	0.3***
FIN	0.21***	NS	NS	NS	NS	NS	0.23***	0.34***	NS
FRA	0.2***	-0.37***	0.24***	NS	0.01*	0.02***	0.16*	0.32***	NS
GBR	0.19***	-0.2***	0.17***	NS	NS	NS	NS	0.22**	0.21***
GRC	0.16***	-0.18***	NS	NS	NS	NS	NS	0.44***	NS
HUN	0.14***	NS	NS	NS	0.01*	NS	NS	0.38***	NS
IRL	0.21***	-0.13***	0.21***	-0.1*	NS	NS	NS	0.32*	0.32**
ISL	0.24***	-0.27***	NS	-0.59***	NS	0.01*	NS	NS	NS
ISR	0.15***	NS	0.15**	N/A	0.02*	NS	0.43***	0.32**	NS
ITA	0.12***	-0.18***	0.14**	NS	NS	NS	0.11*	0.59***	NS
JPN	0.17***	-0.52***	NS	0.15**	NS	NS	NS	0.47***	0.27**
KOR	0.28***	-0.33***	NS	NS	NS	NS	NS	NS	0.37***

LUX	0.13***	-0.16***	NS	NS	NS	NS	NS	0.47*	NS
LVA	0.11***	NS	NS	NS	NS	NS	NS	0.21**	0.18*
MEX	NS	NS	-0.35**	NS	NS	NS	0.17**	NS	NS
NLD	0.11***	-0.28***	NS	NS	NS	NS	NS	0.45**	-0.34**
NOR	0.27***	-0.21***	0.28***	NS	NS	NS	NS	0.24*	0.25**
NZL	0.25***	NS	0.33***	NS	NS	NS	-0.23*	0.34***	0.37***
POL	0.14***	0.08**	NS	NS	NS	NS	NS	0.36***	NS
PRT	0.08***	-0.09***	NS	0.16*	NS	NS	NS	0.24**	NS
SVK	0.13***	NS	NS	NS	NS	0.01**	0.14**	0.25***	NS
SVN	0.14***	NS	NS	NS	NS	NS	0.12*	N/A	NS
SWE	0.26***	-0.21***	0.31***	N/A	N/A	N/A	NS	0.2*	0.22**
TUR	NS	NS	NS	NS	0.01*	NS	NS	0.57***	NS
USA	0.14***	-0.21***	0.15***	NS	NS	NS	NS	NS	0.38***
**SCIEEFF**									
**COUNTRY**	**ESCS**	**GENDER_girl**	**IMMIG_yes**	**SCHLTYPE**	**STRATIO**	**CLSIZE**	**IBTEACH_S**	**TDTEACH_S**	**TEACHSUP_S**
AUS	0.37***	-0.25***	0.11***	NS	NS	NS	0.24***	NS	NS
AUT	0.28***	-0.25***	NS	-0.13*	-0.01**	NS	0.1*	0.22***	NS
BEL	0.27***	-0.29***	0.14**	NS	0.02*	NS	0.44***	NS	-0.17*
CAN	0.32***	-0.26***	0.08**	NS	0.01**	NS	0.21***	NS	0.12*
CHE	0.22***	-0.29***	NS	NS	NS	NS	NS	0.14*	-0.18*
CHL	0.21***	NS	NS	0.17***	NS	NS	NS	NS	NS
CZE	0.22***	-0.18***	NS	NS	NS	NS	0.2***	NS	-0.15*
DEU	0.16***	-0.33***	NS	NS	NS	NS	NS	0.35**	-0.2*
DNK	0.27***	-0.4***	0.19***	NS	NS	NS	0.41***	NS	NS
ESP	0.21***	-0.29***	NS	NS	NS	NS	NS	NS	NS
EST	0.24***	-0.11***	0.11*	NS	NS	NS	NS	NS	NS
FIN	0.3***	-0.27***	-0.22**	-0.15*	NS	NS	0.19*	0.36***	NS
FRA	0.3***	-0.34***	0.15**	NS	NS	0.01*	0.28***	NS	NS
GBR	0.27***	-0.27***	0.13**	-0.16***	0.02***	NS	NS	NS	NS
GRC	0.21***	-0.21***	NS	NS	NS	NS	0.26***	NS	NS
HUN	0.15***	-0.07*	NS	0.13*	NS	NS	0.18*	NS	NS

IRL	0.33***	-0.26***	NS	NS	NS	NS	NS	0.26*	NS
ISL	0.38***	-0.51***	NS	NS	NS	NS	NS	0.37**	NS
ISR	0.22***	-0.24***	0.13*	N/A	NS	NS	0.39***	-0.24*	0.36**
ITA	0.2***	-0.21***	NS	0.15*	NS	NS	0.11*	0.22**	0.11*
JPN	0.27***	-0.25***	NS	NS	NS	NS	NS	0.37***	NS
KOR	0.37***	NS	NS	NS	0.03**	-0.02**	0.15*	NS	NS
LUX	0.2***	-0.23***	NS	NS	NS	NS	NS	0.59*	NS
LVA	0.19***	NS	0.15*	NS	NS	0.01*	0.32***	NS	NS
MEX	0.08***	NS	NS	NS	NS	NS	0.32***	NS	NS
NLD	0.22***	-0.29***	NS	NS	NS	NS	NS	0.33*	NS
NOR	0.37***	-0.18***	0.13*	NS	NS	NS	NS	NS	NS
NZL	0.36***	-0.28***	0.12*	NS	NS	NS	NS	NS	NS
POL	0.24***	NS	NS	-0.24*	NS	0.01**	NS	NS	NS
PRT	0.2***	-0.18***	NS	NS	NS	NS	0.33***	NS	NS
SVK	0.25***	-0.18***	0.41*	NS	NS	NS	0.36***	NS	NS
SVN	0.27***	-0.14***	NS	NS	NS	NS	NS	N/A	0.09*
SWE	0.29***	-0.39***	NS	N/A	N/A	N/A	NS	NS	NS
TUR	0.09***	0.11**	NS	NS	NS	NS	NS	0.27**	NS
USA	0.24***	-0.27***	NS	NS	NS	NS	0.33***	NS	NS
**INSTSCIE**									
**COUNTRY**	**ESCS**	**GENDER_girl**	**IMMIG_yes**	**SCHLTYPE**	**STRATIO**	**CLSIZE**	**IBTEACH_S**	**TDTEACH_S**	**TEACHSUP_S**
AUS	0.14***	NS	0.21***	NS	NS	NS	0.1*	NS	0.15***
AUT	0.09***	0.09***	0.2***	NS	NS	NS	0.15*	NS	0.23**
BEL	0.09***	-0.06*	0.14***	NS	NS	NS	NS	NS	0.17**
CAN	0.17***	0.09***	0.19***	0.09*	NS	NS	NS	NS	0.22***
CHE	0.07***	NS	NS	NS	NS	NS	NS	NS	NS
CHL	NS	0.09**	NS	NS	NS	NS	NS	NS	0.15*
CZE	NS	NS	NS	0.15*	NS	0.01**	0.27***	NS	NS
DEU	0.06**	-0.24***	NS	NS	NS	NS	NS	NS	NS
DNK	0.08***	NS	0.24***	NS	NS	NS	NS	NS	NS
ESP	0.09***	-0.1***	0.1*	NS	NS	NS	NS	NS	NS

EST	0.08***	NS	NS	NS	0.01*	NS	NS	NS	0.18**
FIN	0.18***	NS	NS	NS	NS	NS	NS	NS	NS
FRA	0.14***	-0.16***	0.2***	NS	NS	0.01**	0.25***	NS	NS
GBR	0.12***	NS	0.18***	NS	0.02***	NS	NS	NS	0.26***
GRC	0.09***	-0.14***	NS	NS	NS	NS	0.11*	0.16*	NS
HUN	0.04**	-0.09**	NS	NS	NS	NS	0.32***	NS	NS
IRL	0.15***	NS	NS	NS	NS	NS	NS	NS	NS
ISL	0.12***	NS	0.21*	0.3*	NS	NS	NS	NS	NS
ISR	0.06**	NS	NS	N/A	0.01*	NS	0.24**	NS	0.49***
ITA	0.1***	-0.07**	0.11**	NS	NS	NS	0.12*	0.25**	NS
JPN	0.13***	-0.21***	NS	NS	NS	NS	NS	NS	0.28***
KOR	0.14***	-0.22***	NS	NS	NS	NS	NS	NS	0.19*
LUX	0.08***	NS	NS	NS	NS	NS	NS	NS	NS
LVA	0.05***	-0.09***	NS	NS	0.01*	NS	0.19**	NS	0.14*
MEX	NS	NS	NS	NS	NS	NS	0.15**	NS	0.12*
NLD	0.07*	-0.14***	NS	NS	NS	NS	NS	NS	-0.24*
NOR	0.18***	0.08**	0.25***	NS	NS	NS	NS	NS	NS
NZL	0.12***	NS	0.26***	NS	NS	NS	NS	NS	NS
POL	0.07***	0.1***	NS	NS	NS	NS	0.15*	NS	NS
PRT	0.09***	-0.08**	NS	NS	-0.01*	NS	NS	NS	NS
SVK	0.04**	NS	NS	NS	NS	NS	0.29***	NS	0.1*
SVN	0.08***	NS	NS	NS	NS	NS	0.09*	N/A	NS
SWE	0.13***	NS	0.25***	N/A	N/A	N/A	NS	NS	0.14*
TUR	NS	0.14***	NS	NS	NS	NS	NS	0.18**	0.24***
USA	0.09***	0.09***	0.2***	NS	NS	NS	0.15*	NS	0.23**
**ANXTEST**									
**COUNTRY**	**ESCS**	**GENDER_girl**	**IMMIG_yes**	**SCHLTYPE**	**STRATIO**	**CLSIZE**	**IBTEACH_S**	**TDTEACH_S**	**TEACHSUP_S**
AUS	-0.06***	0.51***	0.1***	NS	NS	NS	0.08*	NS	-0.09*
AUT	-0.09***	0.33***	0.23***	0.09*	NS	-0.01**	NS	NS	NS
BEL	-0.04**	0.48***	-0.08*	0.19***	NS	0.01*	NS	0.24**	NS
CAN	-0.08***	0.59***	NS	NS	NS	NS	NS	NS	NS

CHE	-0.05***	0.42***	0.22***	NS	NS	NS	NS	NS	0.24***
CHL	-0.07***	0.31***	NS	NS	NS	NS	NS	NS	0.14*
CZE	-0.03*	0.36***	NS	NS	NS	NS	NS	NS	NS
DEU	-0.09***	0.39***	0.08*	NS	-0.01*	NS	NS	NS	NS
DNK	-0.1***	0.56***	NS	NS	-0.01**	NS	-0.13*	NS	NS
ESP	NS	0.39***	0.08*	0.08*	NS	NS	NS	0.17*	NS
EST	-0.09***	0.45***	NS	NS	NS	NS	NS	NS	-0.16*
FIN	-0.11***	0.36***	0.31***	NS	NS	NS	0.16**	0.15*	-0.28***
FRA	NS	0.45***	0.12**	NS	NS	0.01**	NS	NS	NS
GBR	-0.06***	0.58***	NS	NS	NS	NS	NS	NS	NS
GRC	-0.07***	0.3***	NS	NS	NS	0.01*	NS	NS	NS
HUN	-0.06***	0.38***	NS	NS	NS	NS	NS	NS	NS
IRL	-0.09***	0.43***	NS	NS	NS	NS	NS	NS	NS
ISL	-0.15***	0.73***	NS	NS	NS	NS	NS	NS	NS
ISR	-0.07***	0.47***	NS	N/A	NS	NS	NS	NS	NS
ITA	NS	0.41***	0.17***	0.22**	NS	NS	-0.15**	NS	0.35***
JPN	NS	0.23***	NS	NS	NS	0.01*	NS	NS	NS
KOR	NS	0.21***	NS	NS	NS	NS	NS	NS	NS
LUX	-0.09***	0.53***	0.09**	NS	NS	NS	NS	NS	NS
LVA	-0.06***	0.33***	NS	NS	NS	NS	NS	NS	-0.13*
MEX	-0.03*	0.31***	NS	NS	NS	NS	-0.13*	NS	NS
NLD	NS	0.34***	0.19**	NS	NS	NS	NS	NS	NS
NOR	-0.1***	0.75***	0.18***	NS	NS	NS	NS	NS	-0.19*
NZL	-0.1***	0.44***	NS	NS	NS	NS	NS	NS	NS
POL	-0.07***	0.39***	NS	NS	0.02**	NS	NS	NS	NS
PRT	-0.05***	0.57***	-0.12*	NS	NS	NS	NS	NS	NS
SVK	-0.03*	0.35***	NS	NS	NS	NS	NS	NS	NS
SVN	-0.06***	0.51***	NS	NS	NS	NS	NS	N/A	-0.07*
SWE	-0.13***	0.63***	0.19***	N/A	N/A	N/A	NS	NS	-0.17**
TUR	NS	0.43***	NS	NS	NS	NS	-0.16**	NS	NS
USA	-0.07***	0.52***	0.13***	NS	NS	0.01*	-0.27***	NS	NS

COOPERATE									
COUNTRY	ESCS	GENDER_girl	IMMIG_yes	SCHLTYPE	STRATIO	CLSIZE	IBTEACH_S	TDTEACH_S	TEACHSUP_S
AUS	0.16***	0.16***	0.12***	NS	NS	NS	NS	0.09**	0.11**
AUT	0.13***	0.45***	NS	NS	NS	NS	NS	0.11***	NS
BEL	0.06***	0.21***	NS	-0.13***	NS	NS	NS	0.21**	NS
CAN	0.15***	0.14***	0.16***	NS	NS	NS	NS	0.13***	NS
CHE	0.07***	0.36***	NS	NS	NS	NS	NS	NS	-0.21**
CHL	0.1***	0.15***	NS	NS	NS	0.01*	NS	0.2**	0.21**
CZE	0.12***	0.24***	-0.14*	NS	NS	0.01*	NS	0.14**	0.11*
DEU	0.11***	0.32***	0.13**	-0.13*	NS	-0.02***	NS	0.18**	NS
DNK	0.12***	0.17***	NS	NS	NS	NS	NS	NS	0.13*
ESP	0.09***	0.17***	0.12**	NS	NS	NS	NS	NS	0.14*
EST	0.11***	0.27***	-0.12**	NS	NS	NS	NS	NS	0.16*
FIN	0.13***	0.27***	NS	NS	NS	NS	NS	NS	NS
FRA	0.16***	0.14***	0.11*	NS	NS	NS	NS	0.16*	NS
GBR	0.1***	0.24***	NS	NS	NS	NS	NS	0.12*	0.18***
GRC	0.04*	0.18***	NS	NS	0.01*	0.01*	NS	0.2**	NS
HUN	0.07***	0.18***	NS	NS	NS	0.01**	NS	0.15*	NS
IRL	0.11***	0.28***	NS	NS	NS	NS	NS	NS	NS
ISL	0.2***	0.08*	NS	-0.36**	NS	NS	NS	0.21*	NS
ISR	0.07***	0.33***	NS	N/A	NS	NS	NS	NS	NS
ITA	0.07***	0.22***	NS	0.14**	NS	NS	NS	0.24***	NS
JPN	0.12***	0.18***	NS	0.08*	NS	0.01***	NS	NS	0.18**
KOR	0.19***	NS	NS	NS	NS	-0.01*	NS	NS	0.19**
LUX	0.07***	0.27***	NS	NS	-0.03**	NS	-0.28*	0.29*	NS
LVA	0.1***	0.23***	-0.14*	NS	NS	NS	NS	0.23**	NS
MEX	0.05***	0.22***	NS	-0.17**	NS	0*	NS	0.16*	NS
NLD	NS	0.11***	0.12*	0.09*	NS	NS	NS	NS	NS
NOR	0.15***	0.36***	NS	NS	NS	NS	NS	NS	NS
NZL	0.12***	0.2***	0.15***	NS	NS	NS	-0.17*	0.18*	0.27**
POL	0.13***	0.09***	NS	NS	NS	NS	NS	NS	NS
PRT	0.07***	0.28***	NS	NS	NS	0.02***	0.13*	NS	NS

SVK	0.12***	0.13***	NS	NS	-0.01*	0.01*	NS	0.1*	NS
SVN	0.1***	0.22***	NS	NS	NS	NS	NS	N/A	NS
SWE	0.15***	0.18***	0.11**	N/A	N/A	N/A	NS	NS	0.16*
TUR	NS	0.15***	NS	NS	NS	NS	-0.25***	0.3**	0.23*
USA	0.14***	0.11***	0.12**	NS	NS	NS	NS	NS	NS
